# Factors Affecting Maternal Respiratory Syncytial Virus Vaccination and the Impact on Infant Hospitalization During the 2023-2024 Season in Dallas, Texas

**DOI:** 10.1177/00333549261434117

**Published:** 2026-05-03

**Authors:** Nicole A. Bailey, Zachary M. Most

**Affiliations:** 1 Division of Neonatal-Perinatal Medicine, Department of Pediatrics, University of Texas Southwestern Medical Center, Dallas, TX, USA; 2 Division of Pediatric Infectious Diseases, Department of Pediatrics, University of Texas Southwestern Medical Center, Dallas, TX, USA; 3 Peter O’Donnell Jr. School of Public Health, University of Texas Southwestern Medical Center, Dallas, TX, USA

**Keywords:** vaccines, child health, ethnic disparities, health care delivery, community health

## Abstract

**Objectives:**

This study assessed factors associated with respiratory syncytial virus (RSV) immunization rates among pregnant women and evaluated the effectiveness of maternal RSV vaccination on infant hospitalization rates.

**Methods:**

We collected data on mothers and their newborns born at a university-affiliated hospital in Dallas, Texas, during the 2023-2024 RSV season if they responded to a telephone survey and confirmed their vaccination status. We used logistic regression to assess associations between demographic factors and receipt of maternal RSV vaccination. We confirmed infant RSV hospitalization by telephone survey and calculated maternal RSV vaccination effectiveness against RSV-associated hospitalization.

**Results:**

From November 1, 2023, through February 29, 2024, 179 of 656 women (27%) received the maternal RSV vaccine. Women aged <30 years were less likely to be vaccinated than older women (*P* ≤ .001). Non-Hispanic White women were more likely to be vaccinated (128 of 332; 39%) than Hispanic (21 of 137; 15%) and non-Hispanic Black (7 of 113; 6%; *P* ≤ .001) women. Women with private health insurance (173 of 462; 37%) were more likely to be vaccinated than women with public (4 of 177; 2%) or no (2 of 17; 12%; *P* ≤ .001) health insurance. Almost 3% of infants born to nonvaccinated women were later hospitalized for RSV infection, whereas none of the infants whose mothers received RSV vaccination were hospitalized due to RSV (vaccine effectiveness = 100%; 95% CI, 14%-100%; *P* = .03).

**Conclusions:**

Demographic disparities existed among women who received maternal RSV vaccination, and RSV vaccination lowered infant RSV hospitalization rates.

Respiratory syncytial virus (RSV), the most common cause of hospitalization among infants in the United States,^1,2^ causes severe lower respiratory tract infection in healthy infants. Each year, 2 to 3 of every 100 infants aged <6 months are hospitalized with RSV,^3^ and 100 to 300 children aged <5 years die of RSV illness.^4,5^ In 2023, new prevention strategies were introduced to protect infants from severe RSV infection.

Palivizumab, a short-acting monoclonal antibody to prevent RSV infection, was licensed by the US Food and Drug Administration (FDA) in June 1998.^6^ Palivizumab is no longer routinely recommended for use.^7^ In August 2023, the Centers for Disease Control and Prevention’s (CDC’s) Advisory Committee on Immunization Practices recommended nirsevimab, a longer-acting monoclonal antibody, for the prevention of severe RSV infection among all infants aged <8 months and among children aged 8 to 19 months at increased risk for severe RSV disease who are entering their second RSV season.^2^ Nirsevimab has a reported 70% to 90% efficacy rate at preventing RSV-associated hospitalization for infants in their first RSV season.^1,2^ Clesrovimab, a newer long-acting monoclonal antibody for RSV protection, was licensed by the FDA in June 2025.^8^

In September 2023, a maternal vaccine to prevent RSV disease in young infants also became available.^2^ Maternal immunization with a single dose of the RSV prefusion F protein (RSVpreF) vaccine (Abrysvo, Pfizer) provides passive immunity against RSV to infants if administered at least 2 weeks before birth.^9,10^ Although other RSV vaccines have been approved for use in older adults, the RSVpreF vaccine is currently the only vaccine recommended by CDC and approved by the FDA for use during pregnancy to prevent severe respiratory illness in young infants.^9,10^ Pregnant women are eligible to receive this vaccine if they are between 32 0/7 and 36 6/7 weeks of gestation during the RSV season and if they do not have a planned delivery within 2 weeks.^9^

During the 2023-2024 RSV season, CDC recommended either the RSVpreF vaccine during pregnancy for mothers or nirsevimab for infants to prevent severe infant RSV illness. During the initial rollout of RSV prevention programs during the 2023-2024 RSV season, many obstetricians in our community prioritized offering the RSVpreF vaccine to all eligible pregnant women. However, demographic disparities in maternal vaccination rates exist and are well documented in public health reports, especially with respect to influenza and diphtheria, tetanus, and pertussis (Tdap) vaccination rates among non-Hispanic Black and Hispanic pregnant women.^11^

Given the novelty of the RSV immunizations, the primary objective of our study was to assess whether any sociodemographic factors could be identified that affect maternal RSV vaccine uptake. We also aimed to estimate maternal RSV vaccine effectiveness against RSV-associated hospitalization among infants aged <6 months.

## Methods

We completed this study in 2 parts: (1) by review of electronic medical records (EMRs) and (2) through a telephone survey. The institutional review boards at the University of Texas Southwestern Medical Center and the Texas Health Presbyterian Hospital Dallas, a university-affiliated hospital (STU-2024-0212), approved the study protocol on March 25, 2024. All research activities complied with ethical regulations and were performed in accordance with regulations of each institution. Informed consent to use data for research purposes was obtained from all patients before data collection. Patients were given the option to refuse to participate by opting out.

This study took place at the Texas Health Presbyterian Hospital Dallas, which is a referral center for level 4 maternal care. This hospital has a level 3 neonatal intensive care unit and an estimated labor and delivery volume of 4000 deliveries per year. The women who delivered at this facility received their obstetrical care from various public health clinics, midwifery services, or private obstetrical providers in the community. Patients were also from diverse socioeconomic, racial, and ethnic backgrounds in Dallas and its neighboring counties.

Based on our aims, we had 2 cohorts of patients for our analyses: (1) women who delivered at the hospital during the study period and (2) newborns who were born to those women. Using EMRs, we identified all newborns born from September 1, 2023, through February 29, 2024. From this group, we included newborns born ≥32 weeks gestational age and their mothers. We excluded newborns born at <32 weeks gestational age; newborns born before September 1, 2023, or after February 29, 2024 (when the RSVpreF vaccine was likely not given); newborns with lethal genetic disorders; and newborns who died in the immediate postpartum period and whose deaths were unrelated to RSV.

From EMRs, we collected the following information on the women: demographic information (including age, race and ethnicity, health insurance status classified by the US Census Bureau^12^), documented breastfeeding status, smoking and drug exposure, multiple gestations, and the date of the RSVpreF vaccination (if given). For newborns, we collected data on gestational age, whether they had siblings, the date of the infant RSV immunization (if given), and hospital readmission due to RSV illness by 6 months of age. We could extract hospital readmission information due to RSV from the EMRs because most hospitals in our large metropolitan area use the same EMR brand. From prenatal records and our state immunization registry that consolidates immunization records from multiple sources, we confirmed the RSV immunization status for most women and infants in this study. When infants reached 6 months of age, we attempted to contact all mothers by telephone to review relevant information. We conducted the telephone survey in English, French, or Spanish. Due to maternal preference, a family member interpreted if the mother did not speak any of these languages. We also communicated with a family member if that person was the legal guardian. On the rare occasion that an infant was born to a surrogate mother or was in foster care, we did not attempt a telephone interview because the legal guardian’s contact information was not provided in the EMR. To determine whether the caregiver provided accurate information, we reviewed information collected from the EMR. We obtained the following information during telephone interviews: infant readmission to hospital due to RSV, date of infant RSV immunization (if given), and date of RSVpreF vaccination (if given). We made 2 separate telephone attempts on nonconsecutive days to reach the caregiver. If attempts were unsuccessful, we excluded the patient from the final analysis.

Because the unavailability of the novel RSVpreF vaccine may have contributed to why so few women were vaccinated before November 2023, we restricted the demographic analysis to women who delivered from November 1, 2023, through February 29, 2024. For the assessment of demographic factors associated with RSVpreF vaccination, we specified each pregnant woman as the unit of analysis and whether the pregnant woman received appropriately timed RSV vaccine as the outcome of interest. Appropriately timed doses were given between 32 0/7 and 36 6/7 gestational weeks and at least 14 days before delivery. We categorized pregnant women who received a dose that was not appropriately timed as not vaccinated. We conducted a sensitivity analysis that excluded all mothers whose infants later received nirsevimab or palivizumab to account for the possibility that the lack of the maternal RSV vaccine in favor of an infant RSV immunization was their planned RSV prophylaxis strategy. For the assessment of vaccine effectiveness, we specified each infant as the unit of analysis, whether the infant’s mother received an appropriately timed dose of RSV vaccine as the exposure of interest, and infant RSV-associated hospitalization up to 6 months of age as the outcome of interest. We excluded infants who received nirsevimab or palivizumab from this analysis. We used the 2-sided Fisher exact test to calculate the *P* value for vaccine effectiveness.

Continuous variables are described with medians and IQRs and categorical variables as frequencies and percentages. Age categories were grouped into 4 equally sized age ranges to prevent sparse data. Univariable analysis and the Pearson χ^2^ test were used to compare categorical variables, which we presented as odds ratios (ORs) and 95% CIs. A multivariable logistic regression model was used to determine independent factors associated with vaccine uptake. Backward stepwise regression was used, including all variables that were significant in the univariable analysis, and we sequentially removed variables with *P* > .10 in the multivariable model. Sensitivity analyses were conducted to estimate the potential effect of misclassification of vaccination status. A similar multivariable model was planned to be used to assess factors associated with risk of infant RSV-associated hospitalization. However, because of the small number of events in the vaccine group, we used the Cornfield method post hoc to calculate unadjusted vaccine effectiveness CIs. We used Stata version 16.1 (StataCorp) to perform statistical analyses.

We used 2-sided *P* = .05 to determine significance. No adjustment was made for multiple comparisons. Based on the available study population size of 1962 mothers and assuming a 40% response rate, we determined that there was 95% power to detect an OR of 2.0 between demographic characteristics if the vaccine uptake was 15%.

## Results

From September 1, 2023, through February 29, 2024, 2000 women delivered at the hospital (eFigure 1 in the Supplement). Few women were vaccinated during the first several months after the introduction of the RSVpreF vaccine. However, vaccination rates increased over time, reaching a peak during January 2024 (eTable 1 in the Supplement).

**Figure 1.**
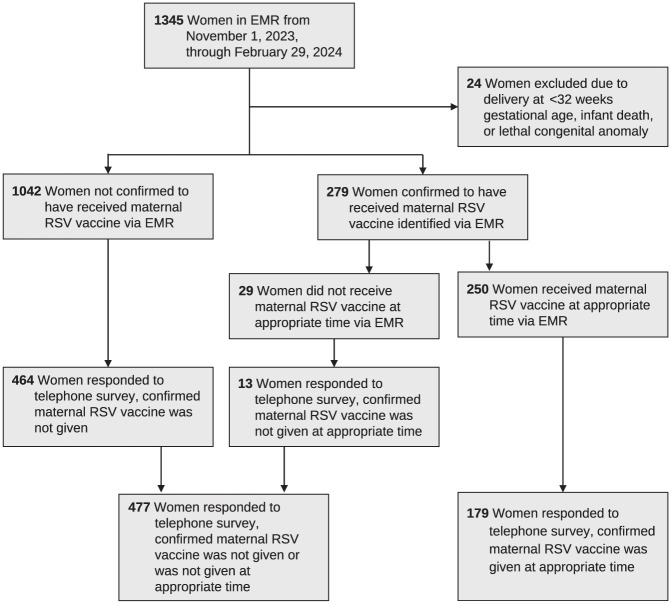
Study enrollment for women who delivered newborns from November 1, 2023, through February 29, 2024, Texas Health Presbyterian Hospital Dallas. Abbreviations: EMR, electronic medical record; RSV, respiratory syncytial virus.

Among 1345 women who delivered at the hospital from November 1, 2023, through February 29, 2024, 24 women were excluded and 656 women responded to our telephone survey. Of the 656 women who responded, 477 (73%) confirmed that they did not receive the RSVpreF vaccine or were not vaccinated at the appropriate time, and 179 (27%) confirmed that they received the RSVpreF vaccine at the appropriate time (Figure 1). Most women were aged 30 to 39 years. Overall, 332 of 656 (51%) women identified as non-Hispanic White, 137 (21%) identified as Hispanic, 113 (17%) identified as non-Hispanic Black, and 57 (9%) identified as non-Hispanic Asian; 462 (70%) had private health insurance, 177 (27%) had public health insurance, and 17 (3%) were uninsured (Table 1).

**Table 1.** Characteristics of women who did and did not receive appropriately timed respiratory syncytial virus (RSV) vaccine before infant birth, Texas Health Presbyterian Hospital Dallas, 2023-2024^a^

Characteristics	Total no. of women (N = 656)	No. (%) of women who received maternal RSV vaccine		Odds ratio (95% CI)	*P* value^b^
		No (n = 477)	Yes (n = 179)		
Maternal age, y					<.001
<20	13	13 (100)	0	0.22^c^ (0.14-0.36)	
20-29	195	172 (88)	23 (12)	0.22^c^ (0.14-0.36)	
30-39	406	261 (64)	145 (36)	1 [Reference]	
≥40	42	31 (74)	11 (26)	0.64 (0.31-1.31)	
Gestational age at birth, wk					.04
32-36	56	47 (84)	9 (16)	0.47 (0.22-0.99)	
37-38	215	152 (71)	63 (29)	1.02 (0.70-1.50)	
39-40	368	262 (71)	106 (29)	1 [Reference]	
41-42	17	16 (94)	1 (6)	0.15 (0.02-1.20)	
Race and ethnicity					<.001
Hispanic	137	116 (85)	21 (15)	0.29 (0.17-0.48)	
Non-Hispanic Asian	57	37 (65)	20 (35)	0.86 (0.48-1.50)	
Non-Hispanic Black	113	106 (94)	7 (6)	0.11 (0.05-0.23)	
Non-Hispanic Other^d^	17	14 (82)	3 (18)	0.34 (0.09-1.20)	
Non-Hispanic White	332	204 (61)	128 (39)	1 [Reference]	
Maternal drug or smoking exposure^e,f^					.02
No	577	411 (71)	166 (29)	1 [Reference]	
Yes	78	65 (83)	13 (17)	0.50 (0.27-0.92)	
Health insurance					<.001
Private	462	289 (63)	173 (37)	1 [Reference]	
Public	177	173 (98)	4 (2)	0.04 (0.01-0.11)	
None	17	15 (88)	2 (12)	0.22 (0.05-0.99)	
Multiple children (N > 1)					.003
No	273	182 (67)	91 (33)	1 [Reference]	
Yes	383	295 (77)	88 (23)	0.60 (0.42-0.84)	
Exclusive breastfeeding					.01
No	315	244 (77)	71 (23)	1 [Reference]	
Yes	341	233 (68)	108 (32)	0.60 (0.42-0.84)	

a Analysis included women who delivered infants from November 1, 2023, through February 29, 2024, and responded to a telephone survey.

b *P* value was calculated using the Pearson χ^2^ test; significance was at α = .05.

c Because there were no mothers in the <20-year age group who received RSV vaccine, the odds ratio was calculated instead for mothers aged <30 years.

d Includes ≥2 races, American Indian or Alaska Native, Native Hawaiian or Other Pacific Islander, or mother declined reporting.

e Includes marijuana; illicit drugs such as cocaine, amphetamines, ecstasy; and use of either tobacco and/or nontobacco products.

f Total patients do not add to 656 because the drug status of 1 mother, who did not get the RSVpreF (RSV prefusion F protein) vaccine, was unknown.

Several demographic factors were strongly associated with receipt of the RSVpreF vaccine in the univariable analysis (Table 1). Women aged <30 years were less likely to have been vaccinated than women aged ≥30 years (OR = 0.22; 95% CI, 0.14-0.36). Women who identified as Hispanic (OR = 0.29; 95% CI, 0.17-0.28) or non-Hispanic Black (OR = 0.11; 95% CI, 0.05-0.23) were less likely to be vaccinated than non-Hispanic White women. Women who delivered preterm (at 32-36 weeks) (OR = 0.47; 95% CI, 0.22-0.99), who had public health insurance (OR = 0.04; 95% CI, 0.01-0.11), or who were uninsured (OR = 0.22; 95% CI, 0.05-0.99) were less likely to be vaccinated than women who delivered at term or who had private health insurance, a finding that was robust to even high rates of misclassification of vaccination status (eTable 2 in the Supplement). In addition, maternal smoking or drug exposure, having other children, and documentation of exclusive breastfeeding were all associated with lower odds of being vaccinated.

In the multivariable analysis, maternal age <30 years, preterm birth, having public health insurance, having other children, and identification as non-Hispanic Black were significantly associated with decreased odds of receiving the RSVpreF vaccine in the primary and sensitivity analyses (Table 2 and eTable 3 in the Supplement).

**Table 2.** Results of multivariable logistic regression model for factors associated with maternal respiratory syncytial virus (RSV) vaccination, Texas Health Presbyterian Hospital Dallas, 2023-2024^a^

Characteristic	Odds ratio (95% CI)	*P* value^b^
Maternal age, y		<.001
<30	0.26 (0.15-0.46)	
30-39	1 [Reference]	
≥40	0.77 (0.35-1.71)	
Gestational age at birth, wk		.02
32-36	0.43 (0.19-0.98)	
37-38	1.05 (0.69-1.61)	
39-40	1 [Reference]	
41-42	0.13 (0.02-1.13)	
Race and ethnicity		<.001
Hispanic	0.81 (0.44-1.48)	
Non-Hispanic Asian	0.81 (0.42-1.56)	
Non-Hispanic Black	0.20 (0.09-0.46)	
Non-Hispanic Other^c^	0.29 (0.08-1.09)	
Non-Hispanic White	1 [Reference]	
Health insurance		<.001
Private	1 [Reference]	
Public	0.08 (0.03-0.23)	
None	0.30 (0.06-1.38)	
Multiple children (N > 1)		.002
No	1 [Reference]	
Yes	0.52 (0.34-0.79)	

a Analysis included women who delivered infants from November 1, 2023, through February 29, 2024, and responded to a telephone survey. Results show backward stepwise logistic regression that initially included all variables that were significant in univariable analysis and removed variables that had *P* > .10 in multivariable analysis.

b *P* value was calculated using the likelihood ratio test; α = .05 was considered significant.

c Includes ≥2 races, American Indian or Alaska Native, Native Hawaiian or Other Pacific Islander, or mother declined reporting.

**Figure 2.**
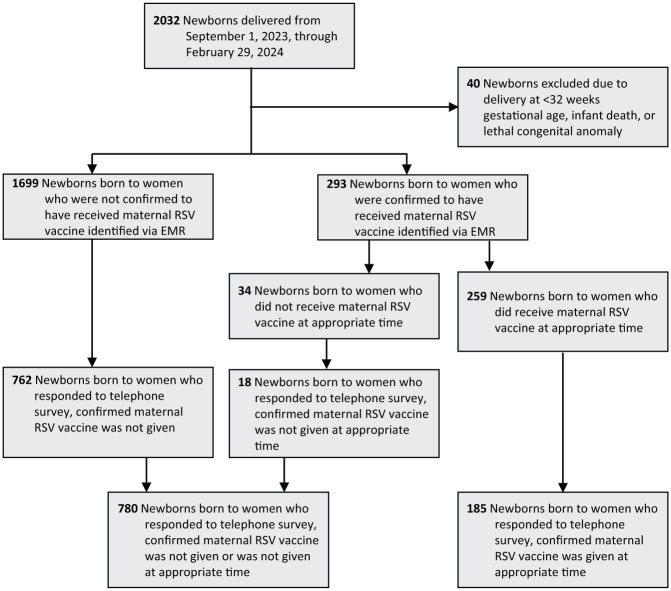
Study enrollment for infants born from September 1, 2023, through February 29, 2024, Texas Health Presbyterian Hospital Dallas. Abbreviations: EMR, electronic medical record; RSV, respiratory syncytial virus.

From September 1, 2023, through February 29, 2024, 2032 newborns were born at the hospital. The number of newborns was higher than the number of women due to multiple gestations. Of these 2032 newborns, 780 had caregivers who responded to the telephone survey and confirmed that the RSVpreF vaccine was not given, and 185 had caregivers who responded to the telephone survey and confirmed that the RSVpreF vaccine was given (Figure 2). When we excluded from the analysis infants who received either nirsevimab or palivizumab, we found that none of the infants born to vaccinated mothers were hospitalized due to RSV (Table 3). Of 623 infants born to nonvaccinated mothers, 17 were hospitalized due to RSV (risk difference of −2.73%; 95% CI, −4.02% to −1.45%). Results showed vaccine effectiveness of 100% (95% CI, 14.0%-100%; *P* = .03).

**Table 3.** Respiratory syncytial virus (RSV)–associated hospitalization rate in infants by maternal RSV vaccine status, Texas Health Presbyterian Hospital Dallas, 2023-2024^a^

Maternal RSV vaccine given	Total no. of infants	RSV hospitalization, no. (%)	
		No	Yes
No	623^b^	606 (97.3)	17 (2.7)
Yes	180^c^	180 (100.0)	0

a Analysis included infants born from September 1, 2023, through February 29, 2024, and who had caregivers respond to the telephone survey.

b 157 newborns who received nirsevimab or palivizumab were excluded from this analysis.

c 5 newborns who received nirsevimab or palivizumab were excluded from this analysis.

When we reviewed RSV hospitalization rates of infants delivered from September 1, 2023, through February 29, 2024, none were hospitalized due to RSV if the pregnant woman received the RSVpreF vaccine, if the infant received any RSV immunization, or a combination thereof (eTable 4 in the Supplement). Other factors independently associated with an increased risk for infant RSV-associated hospitalization were month of birth, preterm birth, and mother not exclusively breastfeeding (eTable 5 in the Supplement).

## Discussion

During the initial rollout of RSV vaccination to pregnant women during the 2023-2024 RSV season, RSVpreF vaccine uptake was low and several demographic factors were associated with a reduced likelihood of receiving the RSVpreF vaccine. Our study showed a maternal RSVpreF vaccine uptake of 27%, which was less than the national maternal RSV vaccine uptake of 32.6% during the 2023-2024 RSV season reported by CDC.^13^ During the 2024-2025 RSV season, CDC reported a maternal RSV vaccine uptake of 38.5%.^14^ Our study demonstrated that almost 3% of newborns born to nonvaccinated mothers were readmitted to the hospital due to RSV illness, which aligned with the national average of 2% to 3% in the United States at that time.^3^ Our study also showed no documented hospitalizations related to RSV infection in infants born to women with adequate maternal RSVpreF vaccination. Currently, the nationwide RSV-associated hospitalization rate among infants is estimated to be <1% due to the initiation and widespread availability of new RSV prevention treatments.^15^

Since the introduction of the maternal RSV vaccine, uptake nationwide was lowest among non-Hispanic Black and Hispanic women, women with public health insurance, women living below the federal poverty level, and women with a college degree or less.^13,14^ Our study demonstrated that RSVpreF vaccination rates were lowest among women who were aged <30 years, had a history of smoking or drug exposure, were multiparous, exclusively breastfed, had public or no health insurance, or identified as non-Hispanic Black or Hispanic. Race, ethnicity, and health insurance coverage are interrelated.^16,17^ In our study, many Hispanic and non-Hispanic Black women either had no health insurance or received public health insurance. Women without health insurance may face cost-related barriers to accessing and paying for the RSVpreF vaccine. In addition, many of the Hispanic and non-Hispanic Black women with public health insurance in our community attended a public health clinic that did not offer the RSVpreF vaccine. According to the Texas Department of State Health Services, underinsured patients must be referred to federally reimbursed locations to receive the RSVpreF vaccine.^18^ This policy might partly explain why RSVpreF vaccination rates were lowest among Hispanic and non-Hispanic Black women and people with public health insurance in our study.

Multiple studies have shown that race and ethnicity are social determinants of health with respect to hospitalization rates for acute respiratory illnesses including RSV.^16,19,20^ Infant RSV hospitalization rates are highest among non-Hispanic Black and American Indian or Alaska Native populations and infants insured by Medicaid.^7,16,21-23^ Furthermore, multiple sociodemographic factors, such as young maternal age, maternal criminal involvement, and maternal history of serious mental health and/or substance use disorders, have been found to be independently associated with increased RSV-related hospital admissions in children.^24^ Therefore, when combined with our findings, it is crucial that local health care systems and public health authorities pursue these groups for RSV prophylaxis.

Our study also showed a correlation between exclusive breastfeeding and decreased infant RSV-associated hospitalization. Exclusive breastfeeding has been shown to be associated with decreased hospitalization rates and health care visits for infants diagnosed with many infections, especially lower respiratory tract infections.^25-27^ Breast milk is protective because it contains antibodies, immune cells, and other factors that help develop the infant’s immune system.^26^ Therefore, promotion of breastfeeding could be a cost-effective strategy to decrease infant RSV-associated hospitalizations in populations with decreased RSV immunization rates.

### Limitations

This study had several limitations. First, our EMR did not always document all immunizations or infant hospitalizations. Thus, it was necessary to call the caregiver to confirm information. If information could not be confirmed, patients were excluded from our analysis, thus lowering our sample size and statistical precision. Selection bias could have been introduced if individuals in some demographic groups received their prenatal care at other locations that were not in our EMR, decreasing the likelihood for these individuals to have EMR documentation of immunization status. However, we attempted to minimize this effect by confirming immunization status with telephone calls. Second, we aimed to call mothers or caregivers within 6 months of birth to confirm information in the EMR. However, this delay may have led to inaccurate responses. Thus, vaccination status of some women may have been misclassified, although unrealistically high differential misclassification rates between demographic groups would have been needed to nullify our results. In addition, through focused questions and verification of information already documented in the EMR, we attempted to minimize misclassification. Third, strategies for determining which mothers would get RSV vaccination versus infant immunization and the availability of the vaccine or immunization shifted throughout the season, which could explain some of the discrepancies observed. Fourth, we may not have been able to address some residual confounders because of lack of available data. Fifth, we evaluated multiple comparisons, which increased the type 1 error rate, meaning that some of the associations may have resulted from random sampling error. Finally, for the RSV vaccine effectiveness outcome, we observed no hospitalizations in the group of infants whose mothers received the RSV vaccine, so we could not calculate vaccine effectiveness with good precision.

Efforts are needed to determine trends in maternal and infant RSV immunization rates during the upcoming years, especially in medically underserved populations. Efforts are also needed to identify reasons why women and infants do not receive RSV prophylaxis (ie, choice vs availability). Educating the public and health care providers on RSV immunization effectiveness, starting conversations during pregnancy to avoid misinformation, and leveraging trusted relationships with pediatricians and obstetricians can all help to improve immunization rates.^28^ Future studies should also compare the length of effectiveness of nirsevimab, clesrovimab, and RSVpreF immunizations in preventing infant RSV hospitalizations.

## Conclusion

Our study showed important demographic disparities in RSV prophylaxis during the initial rollout of the RSVpreF vaccine that need to be eliminated. Our study also demonstrated a substantial benefit of maternal and infant RSV immunizations in lowering infant RSV hospitalization rates. Health care professionals should strive to promote RSV immunization to protect our youngest and most vulnerable patients.

## Supplemental Material

sj-docx-1-phr-10.1177_00333549261434117 – Supplemental material for Factors Affecting Maternal Respiratory Syncytial Virus Vaccination and the Impact on Infant Hospitalization During the 2023-2024 Season in Dallas, Texas Supplemental material, sj-docx-1-phr-10.1177_00333549261434117 for Factors Affecting Maternal Respiratory Syncytial Virus Vaccination and the Impact on Infant Hospitalization During the 2023-2024 Season in Dallas, Texas by Nicole A. Bailey and Zachary M. Most in Public Health Reports®
