# Supplementary material for: Factors Affecting Maternal Respiratory Syncytial Virus Vaccination and the Impact on Infant Hospitalization During the 2023-2024 Season in Dallas, Texas
Source: Public Health Rep. 2026 May 3:00333549261434117. Online ahead of print. doi: 10.1177/00333549261434117 (PMC13139313; doi:10.1177/00333549261434117)
Supplement: sj-docx-1-phr-10.1177_00333549261434117 – Supplemental material for Factors Affecting Maternal Respiratory Syncytial Virus Vaccination and the Impact on Infant Hospitalization During the 2023-2024 Season in Dallas, Texas [file sj-docx-1-phr-10.1177_00333549261434117.docx]

| **eTable 1. Receipt of Maternal RSV Vaccine By Month of Birth** | | | |
| --- | --- | --- | --- |
| Month of Birth | No Confirmed **Maternal RSV vaccine**, n (%) | Confirmed **Maternal RSV vaccine**, n (%) | Total Pregnant Women |
| September 2023 | 140  (100%) | 0  (0%) | 140  (100%) |
| October 2023 | 147  (97%) | 4  (3%) | 151  (100%) |
| November 2023 | 124  (82%) | 28  (18%) | 152  (100%) |
| December 2023 | 128  (74%) | 46  (26%) | 174  (100%) |
| January 2024 | 103  (65%) | 56  (35%) | 159  (100%) |
| February 2024 | 122  (71%) | 49  (29%) | 171  (100%) |
| **Total** | **764**  (81%) | **183**  (19%) | **947**  (100%) |
| RSV – respiratory syncytial virus | | | |

| **eTable 2.** Sensitivity analysis to assess the potential impact of misclassification of vaccination status. | | | | | |
| --- | --- | --- | --- | --- | --- |
|  | **Category** | **Unvaccinated, n (%)** | **Vaccinated, n (%)** | **OR (95% CI)** | **Misclassification rate*** |
| **Observed data** |  |  |  |  |  |
| Race/ethnicity | White,NH | 204 (61.4) | 128 (38.6) | Ref |  |
|  | Hispanic | 116 (84.7) | 21 (15.3) | 0.29 (0.17 – 0.48) |  |
|  | Black,NH | 106 (93.8) | 7 (6.2) | 0.11 (0.05 – 0.23) |  |
|  |  |  |  |  |  |
| **Potential true values** |  |  |  |  |  |
| Race/ethnicity | White,NH | 204 (61.4) | 128 (38.6) | Ref | 0 |
|  | Hispanic | 97 (70.8) | 40 (29.2) | 0.66 (0.43 – 1.01) | 48% |
|  | Black,NH | 81 (71.7) | 32 (28.3) | 0.63 (0.40 – 1.00) | 78% |
|  |  |  |  |  |  |
| **Observed data** |  |  |  |  |  |
| Payor | Private | 289 (62.6) | 173 (37.4) | Ref |  |
|  | Public | 173 (97.7) | 4 (2.3) | 0.04 (0.01 – 0.11) |  |
|  |  |  |  |  |  |
| **Potential true values** |  |  |  |  |  |
| Payor | Private | 289 (62.6) | 173 (37.4) | Ref | 0 |
|  | Public | 125 (70.6) | 52 (29.4) | 0.70 (0.48 – 1.01) | 92% |
| Only misclassification of individuals who were vaccinated by counted as unvaccinated in our data were assessed. The likelihood of unvaccinated individuals being counted as vaccinated was low.  Non-differential misclassification of vaccination status would have biased the observed results towards an odds ratio closer to 1, so we only assessed the potential impact of differential misclassification, with greater rates of misclassification of vaccination status in the comparison rather than the reference groups.  *The misclassification rate is the hypothetical proportion of vaccinated individuals in that category who would need to have been misclassified as unvaccinated in our data to make the 95% confidence interval of the true odds ratio cross 1. These results indicate that if vaccination status was accurately recorded for all NH-White individuals, then vaccination status would have to have been misclassified for 47% of Hispanic individuals for the true odds ratio confidence interval to include 1.  NH – Non-Hispanic | | | | | |

| **eTable 3.** Results of Multivariable Logistic Regression Model for Factors Associated with Maternal RSV Vaccination, Excluding Mothers Whose Children Received Nirsevimab or Palivizumab | | | |
| --- | --- | --- | --- |
| Demographic | Odds Ratio | 95% confidence interval | *P* |
| **Maternal Age (years)** |  |  | 0.0001 |
| Less than 30 | 0.28 | 0.15-0.50 |  |
| 30-39 | Reference |  |  |
| 40+ | 0.84 | 0.36-1.94 |  |
|  |  |  |  |
| **Gestational Age at Birth (weeks)** |  |  | 0.05 |
| 32-36 | 0.36 | 0.14-0.93 |  |
| 37-38 | 1.06 | 0.68-1.66 |  |
| 39-40 | Reference |  |  |
| 41-42 | 0.21 | 0.02-2.00 |  |
|  |  |  |  |
| **Race and Ethnicity** |  |  | 0.0003 |
| White,Non-Hispanic | Reference |  |  |
| Hispanic | 0.85 | 0.44-1.63 |  |
| Black,Non-Hispanic | 0.20 | 0.09-0.47 |  |
| Asian,Non-Hispanic | 1.04 | 0.51-2.11 |  |
| Other* | 0.29 | 0.07-1.13 |  |
|  |  |  |  |
| **Insurance** |  |  | <0.0001 |
| Private | Reference |  |  |
| Public | 0.07 | 0.02-0.21 |  |
| None | 0.12 | 0.02-0.99 |  |
|  |  |  |  |
| **Multiple Children (N>1)** |  |  | 0.006 |
| No | Reference |  |  |
| Yes | 0.53 | 0.34-0.83 |  |
| Results of backwards stepwise logistic regression that initially included all variables that were statistically significant in univariable analysis and removed variables that had *P*>.1 in multivariable analysis. RSV – respiratory syncytial virus.  *includes two or more races, American Indian or Alaska Native, Native Hawaiian or Other Pacific Islander, or declines | | | |

| **eTable 4. RSV-Associated Hospitalization By Maternal or Infant Immunization Status** | | |
| --- | --- | --- |
| RSV Immunizations Given | RSV Hospitalization  No n= (%) | RSV Hospitalization  Yes n= (%) |
| None | 606  (98%) | 17  (2%) |
| Maternal RSV vaccine only | 180  (100%) | 0  (0%) |
| Nirsevimab only | 152  (100%) | 0  (0%) |
| Palivizumab only | 2  (100%) | 0  (0%) |
| Maternal RSV vaccine and nirsevimab | 4  (100%) | 0  (0%) |
| Maternal RSV vaccine and palivizumab | 1  (100%) | 0  (0%) |
| Nirsevimab and palivizumab | 3  (100%) | 0  (0%) |
| **Total** | **948**  **(98%)** | **17**  **(2%)** |
| RSV – respiratory syncytial virus | | |

| **eTable** **5.** Factors Associated With Risk of RSV-Associated Hospitalization in Infants, Excluding Children Who Received Nirsevimab or Palivizumab | | | | | |
| --- | --- | --- | --- | --- | --- |
| Demographic | No. | No. with RSV-associated hospitalization (%) | Crude Odds Ratio (95% CI) | Adjusted Odds Ratio (95% CI) | Adjusted *P* value |
| **Maternal RSV vaccination** |  |  |  |  |  |
| Yes | 180 | 0 (0%) | 0.00 (n/a) |  |  |
| No | 623 | 17 (2.7%) | reference |  |  |
|  |  |  |  |  |  |
| **Month of birth** |  |  |  |  | 0.02 |
| September 2023 | 118 | 7 (5.9%) | Reference | Reference |  |
| October 2023 | 109 | 7 (6.4%) | 1.09 (0.37-3.21) | 1.59 (0.50-5.01) |  |
| November 2023 | 125 | 0 (0%) | 0.00 (n/a) | --- |  |
| December 2023 | 143 | 2 (1.4%) | 0.22 (0.05-1.10) | 0.30 (0.06-1.53) |  |
| January 2024 | 147 | 1 (0.7%) | 0.11 (0.01-0.90) | 0.14 (0.02-1.24) |  |
| February 2024 | 161 | 0 (0%) | 0.00 (n/a) | --- |  |
|  |  |  |  |  |  |
| **Preterm birth** |  |  |  |  | 0.01 |
| No | 738 | 12 (1.6%) | Reference | Reference |  |
| Yes | 65 | 5 (7.7%) | 5.04 (1.72-14.8) | 4.56 (1.40-14.8) |  |
|  |  |  |  |  |  |
| **Maternal age** |  |  |  |  |  |
| <20 years | 16 | 1 (6.3%) | 3.59 (0.43-30.1) |  |  |
| 20-29 years | 245 | 5 (2.0%) | 1.12 (0.37-3.38) |  |  |
| 30-39 years | 493 | 9 (1.8%) | Reference |  |  |
| 40+ years | 49 | 2 (4.1%) | 2.29 (0.48-10.9) |  |  |
|  |  |  |  |  |  |
| **Race and ethnicity** |  |  |  |  |  |
| White,NH | 401 | 6 (1.5%) | Reference |  |  |
| Hispanic | 181 | 5 (2.8%) | 1.87 (0.56-6.21) |  |  |
| Black,NH | 138 | 5 (3.6%) | 2.47 (0.74-8.24) |  |  |
| Asian,NH | 60 | 1 (1.7%) | 1.12 (0.13-9.43) |  |  |
| Other,NH* | 23 | 0 (0%) | 0.00 (n/a) |  |  |
|  |  |  |  |  |  |
| **Insurance** |  |  |  |  |  |
| Private | 560 | 10 (1.8%) | Reference |  |  |
| Public | 221 | 7 (3.2%) | 1.80 (0.68-4.78) |  |  |
| None | 22 | 0 (0%) | 0.00 (n/a) |  |  |
|  |  |  |  |  |  |
| **Maternal smoking/drugs**** |  |  |  |  |  |
| No | 708 | 15 (2.1%) | Reference |  |  |
| Yes | 94 | 2 (2.1%) | 1.00 (0.23-4.45) |  |  |
|  |  |  |  |  |  |
| **Multiple Children (N>1)** |  |  |  |  |  |
| No | 328 | 3 (0.9%) | Reference |  |  |
| Yes | 475 | 14 (3.0%) | 3.29 (0.94-11.5) |  |  |
|  |  |  |  |  |  |
| **Exclusive breastfeeding** |  |  |  |  | 0.02 |
| No | 386 | 14 (3.6%) | Reference | Reference |  |
| Yes | 417 | 3 (0.7%) | 0.19 (0.05-0.68) | 0.22 (0.06-0.79) |  |
| Adjusted odds ratios were calculated using backwards stepwise logistic regression that initially included all variables that were statistically significant in univariable analysis and removed variables that had *P*>.1 in multivariable analysis. Confidence intervals for vaccine effectiveness of maternal RSV vaccination could not be calculated because there were no events in the vaccine group.  *includes two or more races, American Indian or Alaska Native, Native Hawaiian or Other Pacific Islander, or declines  **includes marijuana, illicit drugs such as cocaine, amphetamines, ecstasy, and use of either tobacco and/or non-tobacco products  RSV – respiratory syncytial virus. NH – Non-Hispanic | | | | | |

2000 women in EMR **from September 1, 2023 to February 29, 2024**

**eFigure 1.** Study Enrollment for Women During September 1, 2023 and February 29, 2024

257 women received maternal RSV vaccine at appropriate time via EMR

30 women did not receive maternal RSV vaccine at appropriate time via EMR

183 women responded to phone survey, confirmed maternal RSV vaccine was given at appropriate time

764 women responded to phone survey, confirmed maternal RSV vaccine was not given or was not given at appropriate time

14 women responded to phone survey, confirmed maternal RSV vaccine was not given at appropriate time

750 women responded to phone survey, confirmed maternal RSV vaccine was not given

287 women confirmed to have received maternal RSV vaccine identified via EMR

1675 women not confirmed to have received maternal RSV vaccine via EMR

38 women excluded due to delivery at age <32 weeks gestational age, infant death or lethal congenital anomaly
